# Adaptations, life-history traits and ecological mechanisms of parasites to survive extremes and environmental unpredictability in the face of climate change

**DOI:** 10.1016/j.ijppaw.2020.07.006

**Published:** 2020-07-31

**Authors:** O. Alejandro Aleuy, S. Kutz

**Affiliations:** Department of Ecosystem and Public Health, Faculty of Veterinary Medicine, University of Calgary, Calgary, Canada

**Keywords:** Extreme environments, Climate change, Parasites, Nematodes, Phenotypic plasticity, Local adaptation, Evolutionary history

## Abstract

Climate change is increasing weather unpredictability, causing more intense, frequent and longer extreme events including droughts, precipitation, and both heat and cold waves.

The performance of parasites, and host-parasite interactions, under these unpredictable conditions, are directly influenced by the ability of parasites to cope with extremes and their capacity to adapt to the new conditions. Here, we review some of the structural, behavioural, life history and ecological characteristics of parasitic nematodes that allow them to persist and adapt to extreme and changing environmental conditions. We focus primarily, but not exclusively, on parasitic nematodes in the Arctic, where temperature extremes are pronounced, climate change is happening most rapidly, and changes in host-parasite interactions are already documented. We discuss how life-history traits, phenotypic plasticity, local adaptation and evolutionary history can influence the short and long term response of parasites to new conditions. A detailed understanding of the complex ecological processes involved in the survival of parasites in extreme and changing conditions is a fundamental step to anticipate the impact of climate change in parasite dynamics.

## Highlights

• The adaptations of parasites to extreme environments are relevant to understand parasite dynamics in a changing world.
• Life cycle, plasticity and thermal adaptation are important to effectively anticipate impacts of climate change in parasites.
• The evolutionary history of parasites is a window to study their adaptive capacity to climate change.

## Graphical abstract

**Figure:**
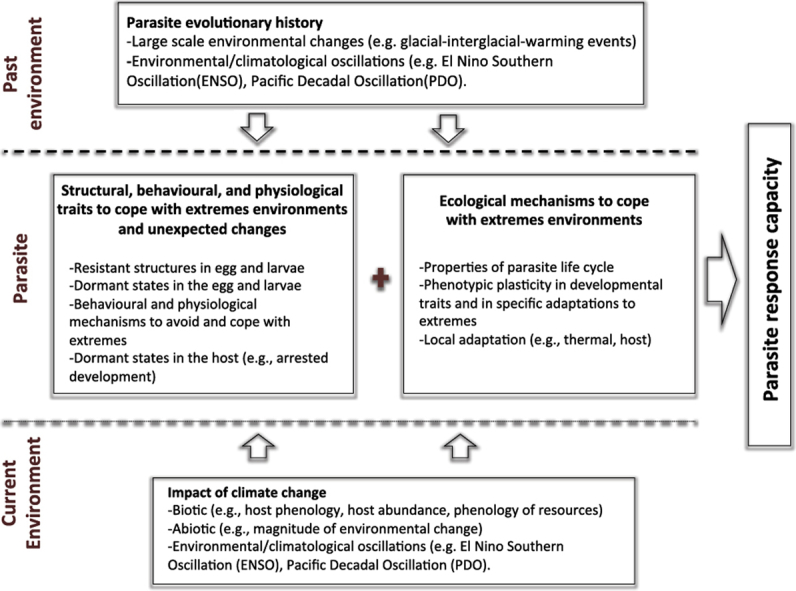

## 1 Introduction

One of the most important health and ecological crises affecting people and global biodiversity is the impact of climate change on the distribution and dynamics of parasites and infectious diseases (Altizer et al., 2013; Anderson et al., 2004; Caminade et al., 2019). Parasites represent at least half of the species diversity of the world, and changes in their diversity, abundance, and distribution have direct implications for public health, the food industry, ecosystem processes and conservation biology (Hatcher et al., 2012; Hechinger et al., 2011; Lafferty, 2009; Pickles et al., 2013; Preston et al., 2016). As the planet warms, climate and weather are becoming increasingly unpredictable. This is manifested by a directional change in temperatures as well as increased frequency and severity of droughts, precipitation, and extremes in both heat and cold (e.g., (Horton et al., 2016; Mann et al., 2017; Naumann et al., 2018; Risser and Wehner, 2017; Schär et al., 2004). The consequences of these environmental changes on host-parasite dynamics and parasite communities, are influenced not just by the degree and patterns of these changes but also by the species-specific capacity of parasites to deal with extremes and new environmental conditions (Deutsch et al., 2008).

Extreme conditions and sudden environmental changes can have a variety of consequences for parasite performance. For instance, if the new conditions are more suitable for the survival of certain individuals, some genotypes are favoured while others disappear, causing changes in allele frequencies in a population via natural selection over multiple generations. However, if individuals are unable to persist or adapt to the changing environment, populations can either disappear or migrate, resulting in a species range to shift (Helmuth et al., 2005). Species-specific adaptations of parasites to conditions, such as freeze and desiccation tolerance, phenotypic plasticity, acclimation capacity, and ecological interactions, can buffer the negative effect of new conditions, increasing the opportunity for persistence and ultimately adaptation. Understanding the subtleties in the life history pathways of parasites that influence the ability of individuals and populations to cope with their abiotic environment is key to effectively anticipate the impact of climate change on host-parasite interactions (Deutsch et al., 2008; Sunday et al., 2011).

Here, we review some of the structural and behavioural adaptations of parasitic nematodes that allow them to survive in extreme conditions, as well as their ecological and life-history traits that can influence their persistence and adaptation to the extreme and unpredictable conditions that may occur as a consequence of climate change. To do this, we focus primarily, but not exclusively, on parasitic nematodes from the Arctic, where climate change is happening most rapidly, temperature extremes are pronounced, and changes in host-parasite interactions are already observed (Kutz et al., 2009). We also include examples of parasitic nematodes from temperate regions where the literature is often more extensive and adaptations may be better understood. Our main goal is to describe and better understand various mechanisms of parasites to survive under extreme conditions and/or sudden changes in temperature, desiccation, and phenological events (e.g., changing seasons). We discuss how these mechanisms may drive and determine the adaptive capacity of parasites to the new conditions and ultimately their distribution at both the local and regional scale as a consequence of climate or other environmental change.

## 2 Nematodes

**Fig. 1:**
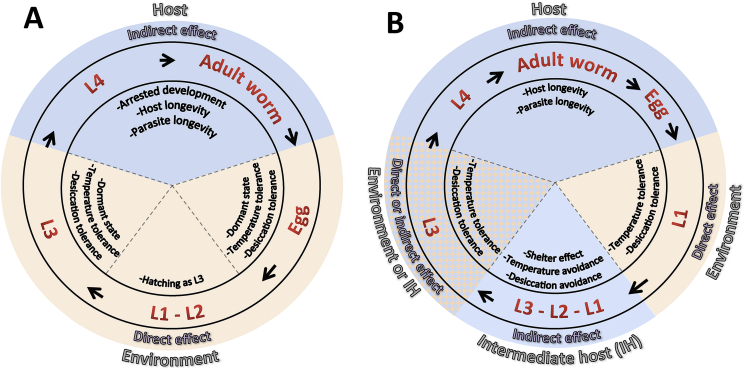
Schematic of two types of life cycles of parasitic nematodes highlighting stage-specific interactions with the environment and hosts, and adaptations to cope with extreme environmental conditions: A) direct life cycle and B) specific indirect life cycle of protostrongylid parasites. In red are indicated the developmental stages of the parasite. The performance (e.g., survival rate, development rate) of developmental stages in the orange area is directly influenced by changes in environmental conditions. Developmental stages in light blue area are indirectly influenced by environmental conditions experienced by the definitive or intermediate hosts. The effect of the environment on the L3 of protostrongylids can be direct or indirect depending if the L3 migrates out of the intermediate host (direct) or if the L3 remains in the intermediate host (indirect). In the inner triangles, examples of stage-specific adaptations to cope with extremes are indicated. (For interpretation of the references to colour in this figure legend, the reader is referred to the Web version of this article.)

Parasitic nematodes are a highly diverse group of organisms (+23,000 species infecting vertebrates), ubiquitous to terrestrial, freshwater, and marine ecosystems (Blaxter and Koutsovoulos, 2015; Castagnone-Sereno and Danchin, 2014; Dobson et al., 2008). They have a variety of energetic costs in the host which translate to direct and indirect negative impacts in agricultural systems, public health, and the ecology and conservation of wild species (Coulson et al., 2018; Hicks et al., 2018; West et al., 2009). Parasitic nematodes develop through different life stages including egg, four juvenile larval stages (L1 [first stage larvae] to L4 [fourth stage larva], with a moult between each stage) and an adult stage. Depending on the species, the development of these stages follows two main life-history pathways. The simplest of which is a direct life cycle, in which the adult worm lays eggs that are passed as eggs, larvated eggs, or L1 in the host feces. Development to the infective third-stage larvae (L3) occurs in the environment either within the egg or as free-living larvae, and the L3 is finally ingested by a new definitive host and develops to the adult worm. A more complex, indirect life cycle occurs for some species in which at least one of the immature stages must occur in an intermediate host (Fig. 1). The effect of environmental conditions on the development and survival of parasitic nematodes varies depending on stage-specific adaptations of eggs and/or larval stages and their interactions with intermediate hosts and the environment (Molnár et al., 2013b). Understanding stage-specific adaptations and species-specific life-history pathways are key to understanding the persistence and dynamics of parasites under changing environmental conditions (Blaxter and Koutsovoulos, 2015; Brooks et al., 2019).

## 3 Structural and behavioural adaptations to cope with extreme conditions and changing environments

### 3.1 Resistant anatomic structures

Species-specific adaptations in eggs and larval stages can enhance individual survival by providing physical barriers to external environmental conditions. A common life-history pathway is to allocate resources into resistant and functional eggshells (Wharton, 1980). Eggshell adaptations can prevent desiccation of the egg in warm temperatures, maintain egg fluids in a liquid state under freezing conditions, and maintain the unhatched larva in a dormant state under unfavourable hatching conditions, among other mechanisms (Lee, 2002; Perry, 1989; Wharton, 1980; Wharton et al., 1993). The eggshell is generally composed of three specialized layers; i) an outer vitelline layer, ii) a middle chitinous layer that is often the thickest, which provides structural strength and acts as a sieve to prevent larger molecules from reaching the deepest layers, and iii) an inner lipid layer that represents the major permeability barrier of the eggshell, and is formed by two or three lipoprotein membranes (Lee, 2002).

When eggs are exposed to extremes in temperature or desiccation, the inner lipid layer interacts with the perivitelline fluid to prevent the egg from desiccating (McSorley, 2003). The capacity of the egg to control fluid permeability differs among species mainly as a consequence of physiological adaptations in the inner lipidic membrane. For instance, the eggs of directly transmitted nematodes like *Haemonchus contortus* and *Trichostrongylus colubriformis* lose water very slowly in highly desiccating conditions. However, *H. contortus* is generally more permeable to water than *T. colubriformis* as its inner lipidic layer contains fewer polar unsaturated lipids and proteins translating in a lower survival under desiccation conditions (Waller, 1971; Waller and Donald, 1970). With respect to freezing temperatures, eggs can promote the conditions to maintain fluids in a liquid state at temperatures below their melting point (i.e. supercooling). The eggshell lowers the freezing point by mobilizing and accumulating different carbohydrates (e.g. trehalose, glycogen) that can reduce water loss and act as cryoprotectants (Ash and Atkinson, 1983; Wharton, 1994). Freeze tolerance of eggs is an important feature for the persistence of *Marshallagia marshalli*, an abomasal nematode endemic to ungulates from arid and very cold environments. Eggs of this species have a 90% survival rate at temperatures of −20 °C for more than 30 days. The eggshell protects not just the embryo, but also larval stages, significantly increasing the parasite's survival under freezing conditions (Box 1) (Aleuy et al., 2020). While the eggs of other parasites from high latitudes like *Nematodirus* spp. (e.g. *N. spathiger* (Marquardt et al., 1959)) are also freeze tolerant, this is not a consistent feature across all Arctic parasites; *Ostertagia gruehneri,* the most common gastrointestinal nematode of *Rangifer tarandus* spp. in high latitudes (Kutz et al., 2012), do not survive freezing but employ other strategies for survival (De Bruyn, 2010; Hoar et al., 2012a).

Box 1 *Marshallagia marshalli* is a nematode infecting the abomasum of free-ranging and domesticated ungulates from Asia, Europe, Africa, North America and South America (Meradi et al., 2011). It is hypothesized that *M. marshalli* originated in Eurasia and dispersed into North America infecting groups of sympatric herbivores inhabiting Beringia during the Pliocene and Pleistocene. During its origin in Eurasia and its expansion to North America, *M. marshalli* was exposed to episodic extreme climatic events including intermittent glaciations, climate fluctuations, different degrees of desiccation, and habitat perturbation (Cook et al., 2017). These extreme conditions and sudden environmental changes are likely to have acted as selecting forces for specific developmental traits that allow the persistence and transmission of *M. marshalli* in extreme environments.

Marshallagia marshalli has a direct life cycle and develops from egg to L3 in the environment exposed to a variety of extreme conditions across its range. In the case of North America for instance, where *M. marshalli* infects wild ungulates like bighorn sheep (*Ovis canadensis*), Dall's sheep (*Ovis dalli*), muskoxen (*Ovibos moschatus*), and caribou (*Rangifer tarandus*), soil temperatures in some areas of its distribution can fluctuate more than 70 °C throughout the year, from ≤ −40 °C in winter to ≥ 30 °C in summer (Hoar et al., 2012b). The free-living stages of this parasite have remarkable and likely interrelated, adaptations to cope with these extreme and changing conditions including i) freeze-tolerant eggs and L3s, ii) development from L1 to L3 that does not rely on the external environment for nutrition, iii) phenotypic plasticity in hatching behaviour that allows hatching as L1 or L3, and iv) a wide thermal developmental range of its free-living stages going from ≤5 °C to 35 °C. *Marshallagia marshalli* eggs are shed throughout the entire year with an increase in shedding intensity during winter months (Aleuy, 2019). Freeze-tolerant eggs are capable of surviving extreme winter conditions and develop to L3 once temperatures increase in the subsequent warmer season. Freeze-tolerant L3 gain importance when hosts undergo seasonal migrations such as with bighorn sheep, Dall's sheep and caribou. Eggs shed in the winter range of the host develop during the warmer season, and the freeze-tolerant L3 completes the transmission in when the host returns to the winter range the following winter. Phenotypic plasticity in hatching behaviour allows rapid developmental adjustment to improve survival depending on short-term environmental cues. While hatching as L1 is the most common hatching behaviour in *M. marshalli*, hatching as L3 is also a possibility with the likelihood of this occurring significantly increasing at high temperatures of development (e.g., 25–35 °C) (Aleuy et al., 2019). This developmental adjustment is in part possible because the L1 and L2 of *M. marshalli* do not ingest nutrients from the external environment during their development to L3. Hatching as L3 protects the more vulnerable L1 and L2, significantly increasing their survival under freezing and desiccation conditions (Aleuy et al., 2020). Finally, the wide range of temperatures to which *M. marshalli* is exposed within its range of distribution in North America translates to a wide thermal developmental range for its free-living stages going from 2 °C to 35 °C (Aleuy, 2019; Carlsson et al., 2013).

Alt-text: Box 1

Once out of the egg, the free-living larvae are more vulnerable to external conditions (Gundy, 1965; Perry and Moens, 2011). The cuticle is a flexible and resistant exoskeleton that is the first line of defence against the environment in the free-living larva of nematodes (Bird, 1954; Page and Johnstone, 2007). The cuticle is mostly proteinaceous with small amounts of lipids and carbohydrates and structurally organized in three main layers (Gundy, 1965). After moulting, some nematode species maintain the moulted cuticle as a cuticular sheath, decreasing the rate of water loss and increasing their survival at high temperatures or desiccating conditions (Allan and Wharton, 1990; Ellenby, 1968). This effect has been explained through different mechanisms, including a decrease in the sheath permeability as the surrounding environment dries (Ellenby, 1968) and the presence of a strongly ionized coat on the surface of the cuticle that facilitates the maintenance of a film of water surrounding the worm, decreasing dehydration (Murrell et al., 1983; O’leary et al., 1998). Ensheathed infective L3 of the Arctic nematode *O. gruehneri* can resist high temperatures of 30 °C for more than 30 days (Hoar, 2012) and those of *M. marshalli* can survive at 35 °C for at least 20 days (Aleuy et al., unpublished observations). Comparable high tolerance of ensheathed larvae to desiccation have been described in plant-parasitic nematodes (Gaur and Perry, 1991) and entomopathogenic nematodes (Menti et al., 1997). Interestingly, the L1 of *Umingmakstrongylus pallikuukensis* and *Varestrongylus eleguneniensis*, two protostrongylid nematodes infecting Arctic ungulates, have no larval sheath, yet have remarkable tolerance to desiccation and extreme freezing temperatures with ~90% survival after being exposed to −40 °C for 180 days (Kafle et al., 2018). The mechanisms driving the freeze tolerance of these nematodes have not been explored, but are likely to be associated with different physiological and biochemical mechanisms well described in other parasitic nematodes, including freeze avoidance, freeze tolerance, and/or the synthesis of different cryoprotectants (e.g., trehalose, glycerol) (Kafle et al., 2018; Wharton, 2011).

### 3.2 Protective behaviours: dormancy and mobility

The free-living stages of parasitic nematodes have evolved a variety of behavioural and physiological mechanisms in which they can delay, avoid or cope with the exposure to extreme conditions. One such alternative is for larvae to enter into a dormant state when facing either seasonal or unpredicted adverse environmental conditions (Sommerville and Davey, 2002). Generally speaking, there are two basic forms of dormancy: diapause, which persists for a set period of time until certain intrinsic changes have been completed, even if the conditions for development are favourable; and quiescence, which is reversible once suitable conditions for development occur (McSorley, 2003; Perry and Moens, 2011). These two forms are often hard to differentiate, as in extreme cases of prolonged quiescence, an intense dormant condition called anabiosis and characterized by metabolic rates below detectable levels, can occur in some species (McSorley, 2003). Similarly, if after diapause the conditions for development are not optimal, the egg/larvae can enter quiescence, making it even more difficult to differentiate both states (Perry, 1989). Dormancy, and quiescence, in particular, are critical physiological mechanisms to cope with predictable seasonal environmental conditions but also enable rapid and effective responses to sudden environmental stress.

In many species, unhatched infective stage larvae can remain dormant within the egg until suitable hatching conditions occur (Eysker, 1993; Gibbs, 1986; Horak, 1981; Perry, 1989; Sommerville and Davey, 2002). Different environmental stresses can trigger this quiescent state but temperature and desiccation are the most frequent causes observed (McSorley, 2003). For instance, *Nematodirus battus* completes its development from egg to L3 in the egg before hatching. Eggs containing L3 can then remain in a quiescent state until temperatures reach a very specific range of 11.5 °C and 17 °C, allowing hatching to occur (Ash and Atkinson, 1983; van Dijk and Morgan, 2008). If eggs are previously exposed to cold winter temperatures, hatching at this range of temperatures increases dramatically, showing how seasonality has modulated this adaptation. It is hypothesized that this nematode has an Arctic origin, and the egg would confer protection to optimize larval survival under extreme freezing conditions, leaving hatching to occur only when the external environmental conditions approach those suitable for transmission (Ash and Atkinson, 1986; Hoberg et al., 2005; van Dijk and Morgan, 2008).

Dormancy can also increase survival for parasites in their intermediate hosts. A higher developmental threshold for L1 of the protostrongylid *Elaphostrongylus rangiferi* may reduce overwinter mortality of larvae in their gastropod intermediate hosts by preventing larvae from starting development unless that development can be completed before winter (Schjetlein and Skorping, 1995). Developing L1 (and L2), have a much lower chance of surviving overwinter than L1 that has not begun development. Gastropods containing developing L1 and L2 also have higher mortality rates than those without developing larvae. Thus, the high development threshold which retains the L1 in dormancy increases the chances of the larvae successfully overwintering in the snail (Schjetlein and Skorping, 1995; Halvorsen and Skorping, 1982).

Dormancy or arrested development is also well-described for larval stages within the host. This is a common feature of several genera of trichostrongyle nematodes infecting the lungs (e.g. *Dictyocaulus viviparus* (Gupta and Gibbs, 1970)), the abomasum (e.g. *Ostertagia ostertagi* (Michel et al., 1975), *Ostertagia gruehneri* (Hoar et al., 2012a,b), *Teladorsagia boreoarcticus* (Kutz et al., 2012), and *Haemonchus contortus* (Berberian and Mizelle, 1957; Blitz and Gibbs, 1972)), and the small intestine (e.g. *Cooperia oncophora* (Bisset and Marshall, 1987; Michel et al., 1974)) of domestic and wild ungulates. These species have a direct life cycle in which the infective L3 is ingested from the pasture and develops through L4 to the adult worm inside the host. Under certain circumstances, the development of a proportion of the L4 ceases for periods of up to several months and, as a consequence, adults do not develop and eggs are not shed during times of the year when conditions are unfavourable for their survival and development in the environment. In populations inhabiting cool temperate areas of the northern hemisphere, this inhibition process occurs over winter, with L4 resuming development to adult parasites the following spring, while in populations from hotter climates this phenomenon occurs in mid to late spring (Armour and Duncan, 1987; Lützelschwab et al., 2005). In both scenarios, these pathways concentrate egg production to seasons where egg survival would be highest (e.g., summer production in temperate and Arctic climates, winter production in hot climates). *Ostertagia gruehneri* infecting the Bathurst caribou herd in Arctic Canada approaches a 100% propensity for winter arrested development (Hoar et al., 2012a). As a consequence, egg production in *O. gruehneri* is highly synchronized to spring and summer when the conditions for development and transmission are more favourable and when the exposure of its freeze-vulnerable eggs to winter conditions is minimum (De Bruyn, 2010; Hoar et al., 2012a). In the case of migratory hosts like caribou, arrested development also synchronizes the parasite infective stage with the host in both time and space (ie., the summer range), helping to maintain parasites in the host population when the transmission is not favoured across all seasons or the entire range of the host (Hoar et al., 2012b).

The capacity of some parasitic nematodes to move in response to external stimuli, like temperature, is of importance for their development and survival (Garrity et al., 2010). Responses of nematodes to temperature start when physical and chemical cues of the environment are processed by the amphids - innervated invaginations usually founded in the anterior region of the nematode (Lopez et al., 2000). These thermal cues influence larval behaviour and migration. For instance, L3 of *H. contortus*, migrate up the blades of grass where the chances of being ingested are greater, but move down the blades seeking more favourable microhabitats when environmental conditions are not conducive to survival (Li et al., 2000; Rees, 1950). This behaviour can play an important role in the survival of free-living larvae of parasitic nematodes in highly seasonal environments, particularly during the transition between warm and cold seasons and vice-versa (i.e. spring and fall), when the odds of extreme oscillations in temperature and humidity in short periods of time is higher.

## 4 Life-history traits and ecological adaptive mechanisms

### 4.1 The paradox of direct and indirect life cycles and the effect of climate change

The life cycle of parasites is a key determinant of the impact of climate change on host-parasite interactions (Kutz et al., 2009). A common theory is that parasites with indirect life cycles will experience a higher risk of extinction under climate change scenarios compared to those with direct life cycles. Parasites with indirect lifecycles require that multiple, precise and obligate interactions occur for successful transmission, and these are more likely to be interrupted in a changing environment. For example, there could be a phenological mismatch between one of the obligate hosts and the parasite or the new conditions may not be suitable for one of the obligate hosts (Dobson et al., 2008; Harvell, 2002; Rohr et al., 2011). Although intuitive, this hypothesis fails to incorporate the idea that intermediate hosts may allow parasites a pathway to persist in environments that would otherwise be unsuitable for survival (Dobson, 1988; Hoberg, 2010). Intermediate hosts are generally more efficient than free-living parasites in moving to microhabitats that modulate ambient climatic conditions, thus allowing parasites to avoid temperature extremes, and, as a function of being inside the intermediate host, humidity extremes are also avoided (Fig. 1). This protective effect due to the thermoregulatory behaviour of its intermediate host is called the ‘shelter effect’ and can increase parasite fitness under certain conditions (https://paperpile.com/c/micx6c/Bvowa; Molnár et al., 2013a). For instance, gastropods like snails and slugs, well known intermediate hosts for a variety of parasites, have strong behavioural responses to diurnal and seasonal environmental conditions (Dainton, 1954; Miles et al., 1931). Both taxa can seek out microhabitats that modulate temperature and humidity extremes, with some species also able to tolerate freezing; snails have the added advantage of being able to aestivate to avoid desiccation and high temperatures (Asahina et al., 1953; Chapperon and Seuront, 2011; Chichester and Getz, 1973; Tewksbury et al., 2008).

The shelter effect has been proposed for the muskox lungworm, *U. pallikuukensis* (Protostrongylidae), and its intermediate host, the slug *Deroceras laeve* (Molnár et al., 2013a; Kutz et al., 2009). In this system, adult parasites reside in the lungs, produce eggs that hatch to first-stage larvae (L1) in the lungs, are carried up the airways and then swallowed and passed in the feces. Development from L1 to the infective third-stage larvae (L3), occurs in slug or snail intermediate hosts. *Umingmakstrongylus pallikuukensi*s occurs in the Canadian Arctic where summer ground surface temperatures can frequently exceed 30 °C. *Deroceras laeve* avoids these temperature extremes through behavioural thermoregulation, moving to cooler microhabitats. Through this behaviour, those slugs parasitized with larvae of *U. pallikuukensis* improve not only their own survival but that of the parasites they contain by avoiding these potentially lethal high temperatures (Kutz et al., 2009). Molnár et al. (2013a) incorporated the shelter effect into a predictive framework using traditional host–macroparasite models complemented with the Metabolic Theory of Ecology (https://paperpile.com/c/micx6c/gwWt4; Molnár et al., 2013b). Resultant models demonstrated two important consequences of the shelter effect, first, a fitness advantage of indirect life cycle over a direct life cycle in high-temperature conditions, and second, a fitness advantage of indirect life cycle over a direct life cycle in highly seasonal environmental settings. This work suggests that in some systems, parasites with indirect life cycles may be buffered from the unpredictable and/or seasonal patterns of extremely warm or cold conditions, and thus may be better equipped to cope with climate change than parasites with direct life cycles.

While not specifically tested by Molnár et al. (2013a) the shelter effect of intermediate hosts likely can be extended to extremes in cold temperatures in highly seasonal environments. For instance, *D. laeve*, overwinters on the Arctic tundra and can survive freezing temperatures of at least −28 °C by producing cryoprotectants that reduce ice formation within the body (Kutz et al., 2002; Storey et al., 2007; Berman et al., 2011). Additionally, *D. laeve* has a multiyear lifecycle that allows multiple overlapping generations throughout the year (Sullivan, 2016; Berman et al., 2011). This consistent slug presence, along with its ability to successfully host protostrongylid larvae during winter, suggests that a relatively constant source of infective larvae may be available to the definitive host throughout the growing season in the Arctic (Sullivan, 2016).

### 4.2 Phenotypic plasticity

Phenotypic plasticity is the capacity of a single genotype to produce different phenotypes or phenotypic responses as a function of the stimulus received from the environment (Bradshaw, 1965; Forsman, 2015; Scheiner, 1993). Phenotypic plasticity is often suggested as one of the mechanisms, along with dispersal and genetic adaptation, by which populations can persist when facing rapid and/or extreme environmental changes (Chevin et al., 2010; Davis et al., 2005). This is particularly true when plasticity occurs in traits important for survival, reproduction, and dispersal, which directly influence the short-term demographic response and also adaptation to the new conditions (Reed et al., 2011). Phenotypic plasticity is central in the context of ecological fitting (i.e. process by which organisms colonize and/or persist in novel environments/conditions (Brooks et al., 2019)) and climate change. Plasticity allows a range of phenotypic responses and/or the expression of historically conserved traits (e.g. exaptations) capable to enhance, or at least maintain, parasite fitness if the phenotypes or traits are suitable for the new conditions. This set of potentially suitable responses to short term environmental changes can increase the window for an adaptive response of the parasite to occur (Brooks et al., 2019; Brooks and Hoberg, 2007).

Phenotypic plasticity in developmental traits is well described in many parasitic nematodes but has rarely been linked to the ability to cope with extreme conditions and changes in climate (Brooks et al., 2019; Tandonnet and Pires-daSilva, 2016; Viney and Diaz, 2011). Phenotypic plasticity can provide physiological, behavioural and ecological short-term responses (i.e. historically conserved capacities) to unexpected weather events and/or extreme seasonal conditions of increasing intensity and/or duration (Agosta et al., 2010; Brooks et al., 2019; Hoberg and Brooks, 2008). For instance, *M. marshalli,* hatches as L1 or L3 depending on environmental cues (Box 1). When developing to L3 occurs in the egg, the eggshell protects the more vulnerable L1 and L2 against external conditions like desiccation and freezing which would otherwise significantly decrease the survival of these stages (Aleuy et al., 2020). The short-term nature (i.e., within the same parasite generation) of this developmental adjustment allows the parasite to rapidly adjust to maximize its survival, transmission and ultimately fitness under adverse conditions.

At the same time, short-term phenotypic responses may buffer the indirect impacts of unpredictable events and climate change on host-parasite interactions. Climate change may influence the rate of contact between helminths and hosts through a variety of indirect mechanisms such as altering host movement and migration due to changes in plant phenology or food sources, among others (Cohen et al., 2018). Phenotypic plasticity, such as the different hatching strategies of *M. marshalli*, may mitigate the impact of temporal and/or spatial mismatch between parasites and hosts by extending the survival of free-living stages in the environment, thus, increasing the chances for contact and ultimately transmission.

Phenotypic plasticity also plays a key role in the adaptive capacity of organisms to the new environmental conditions (Draghi and Whitlock, 2012). Populations can adapt to rapid changes through individuals or genes already present within populations that would increase in their frequency depending on the selective pressures that are a consequence of the changing environment (Jump and Penuelas, 2005; Kelly et al., 2003). The phenomenon of arrested development, commonly described for several species of strongyle parasites in domestic livestock (Armour and Duncan, 1987), but also observed in wildlife, illustrates this. For example, in *Obeliscoides cuniculi,* a nematode infecting the stomach of rabbits, the propensity for arrested development increased from 15 to 90% in response to cold treatment in only 5 generations, with this high propensity to arrested development remaining for the time that the cold treatment was maintained (Watkins and Fernando, 1984). The propensity of arrested development for *O. gruehneri* varies among *Rangifer* populations from the Canadian Arctic, Finland and the subAntarctic island of South Georgia (Hoar et al., 2012a; Hrabok et al., 2006; Leader-Williams, 1980) and also responds quickly to new environmental conditions, suggesting a highly plastic nature of this trait. In caribou from the Canadian Arctic, the propensity for arrested development of the L4 in the abomasum approaches 100% (Hoar et al., 2012a). However, this propensity changed within 3 years when the parasite, sourced from an arctic population, was translocated and established in a captive population of reindeer in southern Canada (Kutz et al., 2012). Similar changes in the proportion of arrested worms depending on the environmental conditions are widely reported for trichostrongyles of domestic livestock (Armour and Duncan, 1987; McKenna, 1973; Michel et al., 1973). The hypothesis that phenotypic plasticity might confer a shortcut to adaptive responses to the new conditions that are a consequence of climate change has been theoretically (Jump and Penuelas, 2005; Kingsolver and Buckley, 2017) and empirically (Kingsolver and Buckley, 2018) tested in different systems but has received very little attention in host-parasite interactions. There is a large body of knowledge regarding the development of parasitic nematodes that can be used to populate evolutionary models and theoretically quantify the importance of phenotypic plasticity in the response of parasite dynamics and host-parasite interactions to extreme environments and climate change. Understanding the phylogenetic and historical context of phenotypic plasticity is of key importance to potentiate the impact of these evolutionary models.

### 4.3 Local thermal adaptation and climate change

The magnitude of variation in daily and seasonal conditions has adaptive consequences on the range of conditions tolerated by organisms inhabiting a given location (Brown et al., 2004; Healy and Schulte, 2012). This basic but informative concept, known as local adaptation, can allow us to better understand and predict the response of organisms to climate change. For instance, near the equator, temperature variation is typically minimal, and the breadth of an organism's thermal tolerance (temperature range limited by the upper and lower thermal range in which organism performance is maximized) is thus predicted to be narrower than that of temperate or arctic species, where extreme daily and seasonal temperature variations are quite common (Clarke, 2003; Deutsch et al., 2008; Sunday et al., 2011). While several studies have examined this prediction and shown correlations between thermal tolerance and geographic distribution in several organisms (Addo-Bediako et al., 2000; Cadena et al., 2012; Deutsch et al., 2008; Healy and Schulte, 2012; Hoffmann and Sgrò, 2011; Huey, 1978; McCain, 2009; Sunday et al., 2011), research on the thermal tolerance of parasites along a latitudinal gradient is sparse (e.g. (Davidson et al., 2008).

**Fig. 2:**
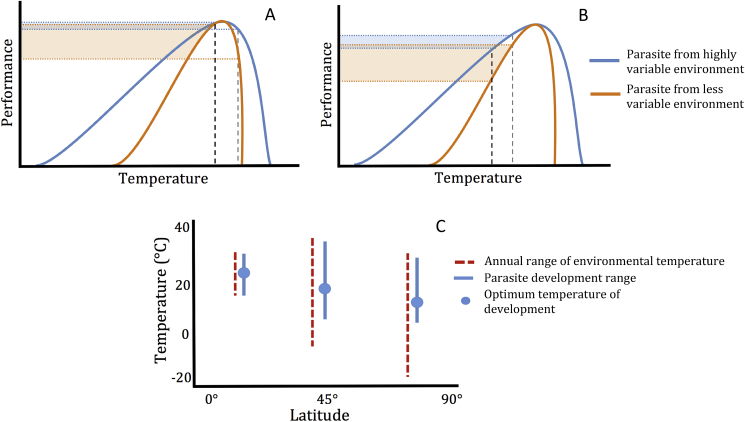
Plots A-B. Hypothetical thermal curves of the free-living stages of two parasite populations with different thermal adaptation histories and similar thermal optimum (highest point in the curve). The blue curve represents a population adapted to a highly variable environment and the orange curve a population adapted to a less variable environment. The dashed black line is a hypothetical current mean temperature in the environment and the dashed grey line represents an increased mean temperature as a consequence of climate change. In plot A, the historical temperature sits close to the thermal optimum in both populations, and an increase in temperature results in a decrease in parasite performance, which is greater for the parasite adapted to the less variable environment. In plot B, the historical temperature is well below the thermal optimum of both parasites, and an increase in temperature results in improved performance for both parasites. In both scenarios, an increase in mean temperature causes a much higher relative change in performance in the population from the less variable environment as indicated in the difference in size among the shade areas. Plot C shows the hypothetical temperature and thermal development ranges for the free-living stages of parasites inhabiting three different latitudes. The temperature range increases with latitude but the development range of parasites does not because, although the thermal range in high latitudes is wider, a large portion of this range occurs <0 °C. While parasites from high latitudes might be highly resistant to freezing temperatures, they are also more vulnerable to high temperatures. (For interpretation of the references to colour in this figure legend, the reader is referred to the Web version of this article.)

According to the above prediction, it is expected that parasite species adapted to environments with narrower daily and seasonal temperature ranges are likely to be more affected by climate change relative to species from ecosystems with wider temperature ranges (Fig. 2A and B) (Deutsch et al., 2008; Dillon et al., 2010; Sunday et al., 2011). To test this hypothesis (Aleuy, 2019), investigated the impact of different climate change scenarios on the performance of the abomasal nematode *M. marshalli* using populations sourced from a latitudinal gradient spanning 25 degrees of latitude in North America. Contrary to the above prediction, the performance of parasites sourced from subarctic regions was more negatively affected by an increase of 2 °C in the mean temperature compared to parasites from temperate regions. Although the absolute temperature range to which parasites are exposed in subarctic regions is substantially wider than in temperate regions, most of that range occurs below zero. This leaves a narrower temperature range above zero in which the parasite is adapted to develop (Fig. 2C). Increases in temperature rapidly pushed the subarctic sourced parasites over their upper thermal limit with a consequent greater negative effect on their performance compared to those sourced from more southern latitudes (Aleuy, 2019). Using a multispecies approach, https://paperpile.com/c/micx6c/IMijt; Grewal et al. (1994) explored the same hypothesis in entomopathogenic nematodes. Although they found differences among species in their thermal range for infection, establishment, and reproduction within locations, they did not find significant differences among conspecifics isolated from different locations. Important to the interpretation of these results is that the nematode strains used in these experiments were maintained in various laboratories for one or more years prior to the study. Laboratory and wild populations can differ substantially, due to founder effects and adaptation to laboratory culture conditions, among others, which makes it difficult to test the hypothesis of local adaptation to their previous conditions (Sternberg and Thomas, 2014).

Local adaptation is not limited to just the parasites, but can also apply to their intermediate hosts, thus adding to the complexity of understanding host-parasite interactions in extreme and changing environments. For example, local thermal adaptation has been demonstrated for gastropods, an important group of intermediate hosts for parasitic nematodes, in marine and terrestrial snail species (Gaitán-Espitia et al., 2013; Marshall et al., 2011; Marshall and McQuaid, 2011; Wood, 1976). Similarly, local adaptation has also been demonstrated for insect vectors of parasites. In *Anopheles* mosquitoes, intermediate hosts of filarial nematodes, the frequency of genetic markers associated with heat and desiccation tolerance decreases with an increase in latitude in central Africa (Cheng et al., 2012).

**Fig. 3:**
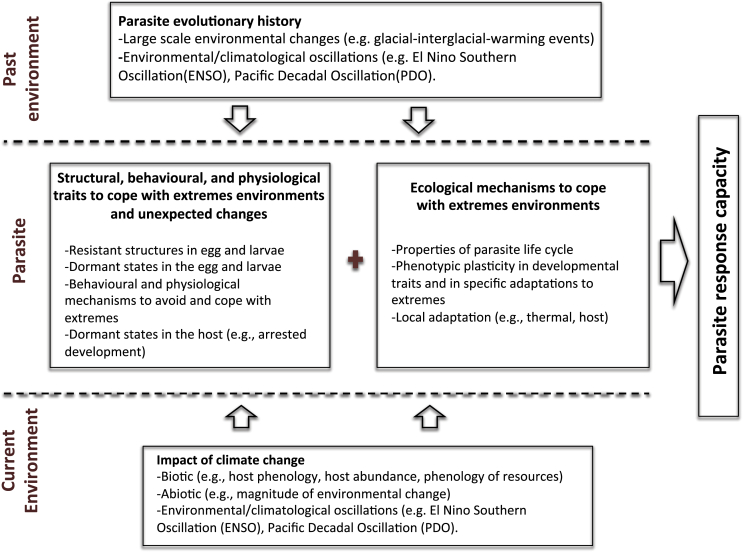
Flow chart outlining factors that can influence the response of parasites to climate change.

The genetic variability of parasites and their vectors/intermediate hosts, and potential for their adaptation in response to long-term environmental changes, are often neglected in research on host-parasite dynamics and climate change, yet these are crucial pieces of the puzzle. If species are highly adapted to local climate conditions, and these conditions are stable, their capacity to shift towards a new climate signal or ‘normal’ may be limited simply due to an absence of genetic variability to cope with variable conditions (Fig. 3). In practical terms, predictive models in disease ecology and epidemiology frequently rely on the basic reproductive number (R_o_) to characterize parasite transmission. In its calculation, R_o_ depends on parasite and vector traits that are highly sensitive to temperatures, such as parasite and vector mortality, and parasite and vector density. Thus, small differences in the shape of thermal response curves between populations can potentially add up across traits and have important consequences for R_o_ (Fig. 2). The interaction of host-parasite systems across variable environments can manifest as both phenotypic plasticity and adaptive responses, which are expressed at the population or individual levels (Pickles et al., 2013). Consequently, to improve our ability to understand host-parasite responses, predictive models need to incorporate the variability associated with the impact of climate variations on specific life-history traits of both hosts and parasites, even among conspecific populations.

### 4.4 Parasite longevity and fecundity

The fecundity and longevity of parasites are important buffers that can influence their persistence under adverse conditions as well as facilitate invasion into new environments in response to changing environmental conditions. This may be even more pronounced for parasites infecting long-lived hosts or hosts with high dispersal capacity. Differential range expansion of two Arctic protostrongylids, *Umingmakstrongylus pallikuukensis* and *Varestrongylus eleguneniensis,* that infect arctic ungulates exemplifies this. Congruent with warming climatic conditions and relaxation of thermal barriers, these two parasites have invaded the Arctic archipelago and significantly expanded their range and abundance northwards. It was originally hypothesized that the rate of expansion would be greatest for *V. eleguneniensis* because it has two hosts, both muskoxen and caribou, the latter host of which is a highly mobile migratory species. In contrast, is a one host-parasite, only infecting muskoxen. The opposite occurred, however, with *U. pallikuukensis* expanding more rapidly and with a higher prevalence and intensity compared to *V. eleguneniensis,* while the rate of expansion of the latter parasite has lagged behind its potential thermal niche (Kafle, 2018). The high fecundity and long lifespan of *U. pallikuukensis* (1000s of larvae shed per gram of feces and a parasite lifespan that may be equivalent to that of the muskox) versus *V. eleguneniensis* (10s of larvae per gram of feces and a predicted lifespan of only months to a few years) are thought to be the major drivers accounting for these differences in abundance and invasion rates (Kafle et al., 2017; Kutz et al., 2001). Lifespans in the realm of years versus months can allow a parasite to persist over years where conditions are not suitable for transmission, thus buffering uncertainties and extremes from seasonal patterns and from relatively short-term shifts in climatic regimes like “El Niño Southern Oscillation” (ENSO) and the Pacific Decadal Oscillation (PDO) (Fig. 3).

## 5 Lessons from evolutionary history

Episodic and extreme changes in climate are a common component in the evolutionary history of many organisms, including parasites. High latitude ecosystems, for instance, have been facing episodic glacial-interglacial-warming events since the late Pliocene and throughout the Pleistocene, challenging the adaptive capacity of the species inhabiting these regions (Cook et al., 2017). While many species were driven to extinction by these relatively rapid changes in climate, others were capable to cope, expand or shift their ranges/hosts, and ultimately rapidly adapt to the new and/or variable conditions (Hoberg et al., 2012). Many of these species are part of the faunal assemblages populating high latitudes today and remain a valuable source to better understand the role that developmental adaptations to extremes can play in the adaptive capacity of parasites to the current rapid environmental changes (Brooks and Hoberg, 2007). For instance, *M. marshalli* and *Nematodirus* spp., despite being phylogenetically different nematodes, share remarkable similarities in their developmental adaptations to cope with extremes (e.g., eggs highly tolerant to freezing and desiccation, development to L3 in the egg) (Hoberg et al., 2005). This suggests common evolutionary forces shaping the adaptive capacity of these parasites to extreme conditions. Also, shifts in climatic regimes like ENSO and the PDO can also influence the persistence of parasites and their evolutionary history (Hoberg et al., 2017). The global mean temperature is directly influenced by the intensity of the ENSO, causing cascade effects in biological systems worldwide (Stocker et al., 2014). Understanding the temporal and spatial dimensions of the evolutionary history of parasites well adapted to extremes, including details like phylogeography, hosts and host shifts, and the climate oscillations driving their recent and distant evolutionary history can deliver key insights to better understand and predict the consequences of climate change on host-parasite interactions (Fig. 3) (Cook et al., 2017; Hoberg et al., 2012; Hoberg and Brooks, 2015).

## 6 Conclusions

A wide range of empirical evidence and modelling approaches on host-parasite interactions demonstrate that climate change is driving range shifts (Kafle, 2018; Kutz et al., 2009; Pickles et al., 2013), increases in abundance and presentation (Hernandez et al., 2013; Laaksonen et al., 2010; Rose et al., 2015; van Dijk et al., 2008), and seasonal changes in phenology (Molnár et al., 2013b; van Dijk et al., 2008) of parasitic nematodes. In addition to a directional change in conditions, a major challenge imposed by climate change on host-parasite interactions is increasing unpredictability of weather, with more intense, frequent and longer extreme events including droughts, precipitation, and both heat and cold waves. To anticipate the consequences of climate change on host-parasite interactions it is necessary to consider the adaptations, life-history traits and ecology of parasites to cope with extremes and unpredictable environmental changes (Fig. 3). The stage-specific nature of these adaptations requires a detailed understanding of the life history of parasites, and their definitive and intermediate hosts, scaling down in some cases to understand differences among distant populations within the same parasite and host species. These adaptations interplay with each species-specific ecological setting to buffer the impact of extremes and unpredictable conditions. In this context, the role of complex life-history and ecological traits, such as the presence of intermediate hosts, phenotypic plasticity in developmental traits, local adaptation to key environmental factors, and parasite fecundity and longevity need to be considered in the host-parasite interaction arena. Finally, the evolutionary histories of both parasite and host offer a window to understand how these adaptations were generated by, or interacted with, past events of global climate change that are similar in many ways to what we are facing today (Brooks et al., 2019; Hoberg et al., 2012). Empirical and theoretical evidence demonstrates that we are facing complex responses in host-parasite systems as a consequence of changes in climate. Incorporating species-specific adaptations, ecological characteristics in life-histories, and evolutionary history in the discussions of climate change and parasite dynamics provides a critical theoretical foundation on which to develop testable hypotheses and predictions, and thus, direct future studies and applied responses.

## Declaration of competing interest

There are no conflict of interest to declare for the manuscript entitled “Climate change and the ecological, behavioural and physiological mechanisms of parasites to survive in the face of environmental unpredictability and extremes”, authored by O. Alejandro Aleuy and Susan Kutz.
